# Outcome after isolated medial patellofemoral ligament reconstruction is dependent on age but not on body mass index or gender

**DOI:** 10.1002/jeo2.70332

**Published:** 2025-07-29

**Authors:** Olivia Bohe, Antonia Schneider, Armin Runer, Sebastian Siebenlist, Andrea Achtnich

**Affiliations:** ^1^ Department of Sports Orthopaedics Technical University of Munich Munich Germany; ^2^ Department of Trauma Surgery Technical University of Munich Munich Germany

**Keywords:** functional outcome, gender specific differences, patellofemoral instability, PROMs, MPFL reconstruction

## Abstract

**Purpose:**

Medial patellofemoral ligament (MPFL) reconstruction is the most used surgical technique in the treatment of patellofemoral instability. However, the role of patient specific factors like age, sex and body mass index (BMI) at surgery is being increasingly discussed. The aim of this study was to study the influence of these factors with regards to functional outcomes and redislocation rates.

**Methods:**

All patients with patellofemoral instability, who were treated with isolated MPFL reconstruction surgery between 01/2017 to 01/2022, were included. Patients with pathologic risk factors, high‐grade cartilage damage, prior surgeries and age <14 years were excluded. Demographic information and information concerning surgery, complications and history were collected. Patient reported outcome measures (PROMs) were collected preoperatively, after 6 and 12 months postoperatively and at final follow‐up using multiple standardised scores (knee injury and osteoarthritis outcome score, International Knee Documentation Committee [IKDC], Tegner activity scale, Kujala, BANFF).

**Results:**

Of the 62 patients included in this study 42 (67.7%) were female with a mean age of 24.8 ± 7.6 years and a mean BMI of 24.5 ± 4.7 kg/m^2^ at the time of surgery. Final follow‐up was 42.3 ± 23.4 months. Fifty‐four (90.3%) patients were satisfied with the functional outcome, four (6.5%) patients suffered recurrent dislocation.

Overall, the functional outcome was very good in our study population (e.g., Kujala 87.0 ± 10.5, IKDC 76.4 ± 13.7). In the subgroup analysis, there were no significant differences in the functional outcome between women and men (e.g., Kujala score: 87.2 ± 11.4 vs. 86.4 ± 7.8, *p* = 0.81) and there was no correlation with BMI at time of surgery (e.g., Kujala, *r* = 0.11, *p* = 0.53). However, statistically significant correlations were detected in functional outcome with the age at surgery.

**Conclusion:**

Older age at the time of surgery has a highly significant negative correlation with the functional outcome after isolated MPFL reconstruction. Therefore, surgeons must be highly vigilant and identify high‐risk patients even before surgery and necessary MPFL reconstruction should not be delayed.

**Level of Evidence:**

Level III, retrospective cohort study.

**Abbreviations**
ADL: activity of daily life
BMI: body mass index
CDI: Caton‐Deschamps index
IKDC: International Knee Documentation Committee
KOOS: knee injury and osteoarthritis outcome score
MPFL: medial patellofemoral ligament
PASS: patient‐acceptable symptomatic state
PROM: patient reported outcome measure
QoL: quality of life
TAS: Tegner activity scale
TTTG: tibial tuberosity‐trochlea groove
VAS: visual analogue scale

## BACKGROUND

With the incidence reported to be as high as 43 per 100,000 in the adolescent population chronic patellofemoral instability is a common problem especially in young patients [15, 21]. When no significant bony risk factors are present, it has been shown that surgical treatment with isolated MPFL reconstruction shows favourable outcomes compared to conservative treatment [21, 23, 26]. The medial patellofemoral ligament (MPFL) serves as the primary restraint against lateral patellar translation and commonly ruptures with traumatic patellar dislocation [2].

While isolated MPFL reconstruction generally yields good clinical results, variability in postoperative success suggests that patient‐specific factors, such as age, body mass index (BMI), and sex, may influence surgical outcomes and recovery trajectories. However, the results presented in current literature are inconclusive.

Patellar dislocations most commonly occur in the second decade of life, with younger patients facing an even higher risk of recurrence [2, 34]. Recently it was suggested that age at surgery, but not at the time of first dislocation, is associated with low outcome scores after MPFL reconstruction as well as trochlear plasty [10, 28].

Concerning BMI, higher BMI is associated with lower preoperative patients reported functional scores. However, most studies suggest, that the postoperative functional outcome and the rate of redislocation or complication does not correlate with patients' BMI [24, 25, 32].

When looking at the patients' sex, it is well known that women are at a higher risk to suffer from patellar dislocation than men [7, 34]. In recent literature, there has been an increased focus on gender‐specific differences in knee anatomy, as well as variations in epidemiology and outcomes following MPFL reconstruction. While differences in knee anatomy have been extensively studied—particularly concerning total knee arthroplasty and gender‐specific requirements—the surgical technique for MPFL reconstruction remains the same for both women and men [3, 14]. Recently, gender specific differences have increasingly moved into focus of various researchers. One systematic review found, for example, that women have a significantly higher risk of major complications, such as recurrent instability or knee joint stiffness [16]. Contrarily, there is also literature suggesting that female sex is not associated with higher rates of recurrent instability or lower outcome [2, 13].

These contradictory results in recent literature warrant further investigation into the role of patient‐specific factors. Clearer research will help orthopaedic surgeons identify high‐risk patients before surgery and tailor treatment plans accordingly.

The aim of this study was to evaluate the influence of these patient‐specific factors on the patient‐reported postoperative outcome of patients with isolated MPFL reconstruction in patellofemoral instability.

## MATERIALS AND METHODS

### Data collection

Approval by the ethics committee was obtained (Study No. 2022‐193‐S‐NP). The study complied with the Declaration of Helsinki and its respective amendments. All patients signed informed consent forms for participation in this study.

In this retrospective study, all patients who were treated operatively with isolated MPFL reconstruction surgery for patellofemoral instability between January 2017 and January 2022 were included. We excluded all patients who were younger than 14 years of age at the time of surgery. Patients with concomitant ligamentous or high‐grade chondral injuries (ICRS grade III or IV) or previous surgical ipsilateral knee interventions were excluded. In order to control possible confounders for outcome, we excluded patients with high‐grade patellofemoral risk factors, namely elevated tibial tuberosity—trochlea groove (TTTG)—distance (>25 mm), severe trochlear dysplasia (Dejour C and D), patella alta (Caton‐Deschamps index [CDI] > 1.3) and severe valgus malalignment (>5°) or rotational deformity of the femur (ante torsion > 25°). Ante torsion was measured on axial MRI images by first defining the femoral neck axis, drawing a line through the centre of the femoral head and neck on a proximal slice. The femoral condyle axis was then determined by connecting the most posterior points of the medial and lateral condyles on a distal slice.

The demographic data of the patients, including age, sex, date of injury, date of magnetic resonance imaging (MRI), date and type of treatment, were retrieved from the electronic medical reporting system. Normal BMI was defined by a BMI up to 24.9, high BMI with a BMI ≥ 25.0.

Preoperatively, patient reported outcome measures (PROMs) were collected using the knee injury and osteoarthritis outcome score (KOOS), International Knee Documentation Committee (IKDC)‐score, Tegner activity score (TAS) and the visual analogue scale (VAS). Six and 12 months postoperatively follow‐up assessment via online questionnaire was conducted using KOOS, IKDC, TAS, VAS and Kujala score.

For the final follow‐up interview patients were contacted via telephone and the questionnaires were collected online at the time of the study (July 2024 to September 2024) utilising KOOS, IKDC, TAS, VAS, Kujala and BANFF‐score. Added were questions on patient satisfaction and recurrence of dislocation up until follow up.

Isolated MPFL reconstruction was performed in patients with recurrent patellar dislocation or instability, after high‐grade patellofemoral risk factors had been ruled out as the cause for the instability and conservative treatment had failed. Surgery was performed by a team of experienced sports orthopaedic surgeons (*n* = 7) in a specialised university hospital department. Surgery was performed using either hamstrings tendon autograft or quadriceps tendon flaps by surgeons' preference. After diagnostic knee arthroscopy, the hamstring tendon autograft is harvested in the usual technique. Over a 2 cm long incision over the medial facet of the patella, tangential insertions of two guide wires into the patella at the medial‐proximal corner follow. Over‐reaming with a 4.0 mm drill, then attaching the hamstring tendon graft to two anchors (i.e., 4.75 mm SwiveLock, Fa. Arthrex). The tendon is then shuttled between the second and third layer of the medial capsule complex. The femoral attachment of the tendon is marked with a guide wire under fluoroscopy anterior to the posterior femoral cortex, distal to the origin of the medial condyle and proximal to the most posterior point of Blumensaat's line. The graft was then shuttled through the prepared tunnel and fixed with an interference screw at 20° of knee flexion with the lateral patellar border level to the lateral border of the trochlear groove. For a detailed description of surgical technique of quadriceps tendon flap we would like to refer to Runer et al. [18].

All patients followed an identical postoperative rehabilitation protocol. Weight‐bearing was restricted to 20 kg on the injured side for the first 2 weeks, after which a gradual increase in weight‐bearing was permitted. The range of motion was limited to 90 degrees of knee flexion for 6 weeks postoperatively. Full active range of motion was permitted starting seven weeks postoperatively, with continuous sports activities (such as cycling and freestyle swimming) allowed after 8 weeks.

### Statistical analysis

The data were analysed using the Statistical Package for the Social Sciences (SPSS, version 27; IBM Inc.). Patients' characteristics were summarised as means and standard deviations (SD) for continuous variables, while categorical variables were presented as absolute numbers and percentages. Prior to further analysis, data normality was tested using Kolmogorov–Smirnoff‐Test. Significance testing was performed using paired *t*‐tests or Mann–Whitey *U* test, as applicable. A post hoc power analysis was performed to assess the differences in outcome scores dependent on the factors, with a significance level set at 0.05. The calculated power was 79.6% (G*Power Version 3.1.9.6). For metric variables Pearson Test was conducted and (*r*) is given as the correlation coefficient. Correlation coefficients of ±0.7 to 1.0 were interpreted as very strong correlation, between ±0.69 to ±0.50 as strong correlation, ±0.49 to ±0.20 as moderate correlation and a coefficient of <±0.2 as negligible correlation [22].

The patient‐acceptable symptomatic state (PASS) and the clinical important difference (CID) were used to evaluate patient satisfaction [29]. The PASS for the IKDC have been reported at 65.5, the KOOS symptom 80.4, KOOS pain 84.7, KOOS ADL 99.3, KOOS sport 57.5, KOOS QoL 53.1 and Kujala at 83.5 [29].

A *p*‐value of <0.05 (two‐sided) was considered statistically significant.

## RESULTS

### Demographic data

In this study, we included 62 patients, 42 (67.7%) being female. The mean age at the time of surgery was 24.8 ± 7.6 years and the mean BMI was 24.5 ± 4.7 kg/m^2^.

A hamstrings tendon autograft was used in 60 (96.8%), while two (3.2%) patients were treated with a quadriceps tendon flap. There were no perioperative complications documented.

We were able to include 62 of the eligible 85 patients for this study with a loss to follow‐up of 26.2%.

### Follow‐up

Mean time from surgery to final follow‐up was 42.3 ± 23.4 months. Six (9.7%) patients reported to be dissatisfied with the surgery and outcome in a yes and no question, with four (6.5%) patients suffering from another dislocation after surgery. 61.5% (*n* = 38) of the cohort reached or exceeded the PASS for the Kujala score, 76.9% (*n* = 48) for the IKDC score.

**Figure 1:**
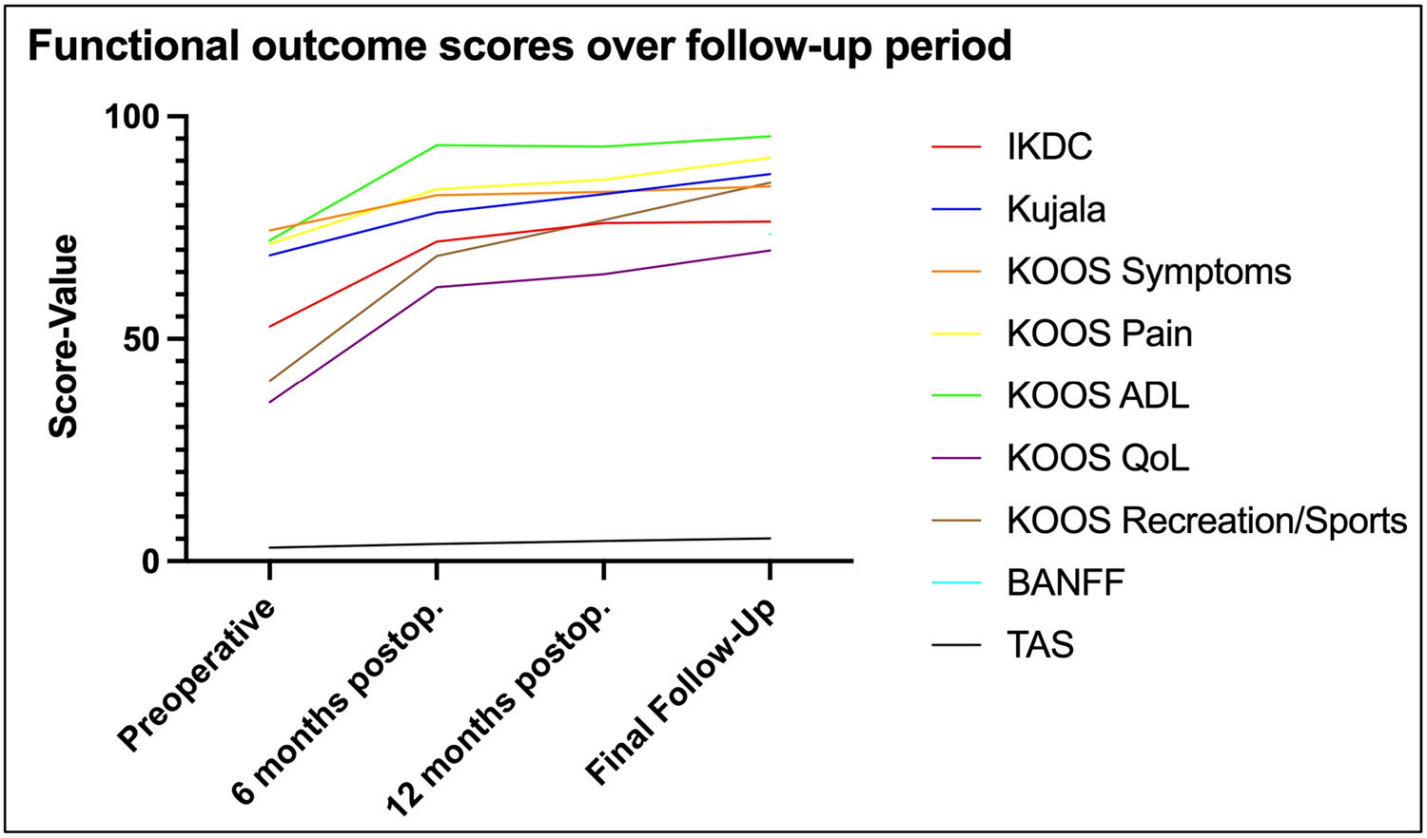
Line plot showing the development of the separate functional outcome scores from preoperatively, over 6‐ and 12‐months postoperatively to the patient reported outcome measures at final follow‐up. ADL, activity of daily life; IKDC, International Knee Documentation Committee; KOOC, knee injury and osteoarthritis outcome score; QoL, quality of life; TAS, Tegner activity scale.

Figure 1 shows the development and increase of the separate functional outcome scores over the follow‐up period in the total study population.

### Comparison by sex

None of the functional scores differed significantly between female and male patients preoperatively or at 6‐ or 12‐month follow‐up. Additionally, there was no statistically significant difference in the percentage of patients who reached outcome above the PASS.

At the final follow‐up, there was also no statistically significant difference in the functional scores between female and male patients. See Table 1 for patient reported functional scores at final follow‐up.

**Table 1:** Functional outcome scores, compared at final follow‐up between female and male patients.

Score	Female	Male	Significance
IKDC	76.5 ± 13.7	75.9 ± 14.6	*p* = 0.91
	Above PASS		
	75.9%	80.0%	*p* = 0.79
Kujala	87.2 ± 11.4	86.4 ± 7.8	*p* = 0.81
	Above PASS		
	65.5%	50.0%	*p* = 0.46
KOOS total score	88.5 ± 10.3	90.2 ± 5.3	*p* = 0.50
KOOS symptoms	85.4 ± 11.1	81.1 ± 16.6	*p* = 0.46
KOOS pain	90.0 ± 12.3	92.6 ± 4.0	*p* = 0.33
KOOS ADL	95.1 ± 8.4	96.7 ± 3.9	*p* = 0.42
KOOS QoL	67.8 ± 25.4	75.8 ± 17.6	*p* = 0.29
KOOS recreation/sports	84.5 ± 18.1	87.0 ± 12.3	*p* = 0.63
TAS	4.9 ± 1.5	5.5 ± 1.7	*p* = 0.18
BANFF	73.0 ± 18.8	74.7 ± 23.5	*p* = 0.50

*Note*: Additionally, for Kujala and IKDC score the percentage of patients who reached scores above PASS and statistical significance is noted. Abbreviations: ADL, activity of daily life; IKDC, International Knee Documentation Committee; KOOS, knee injury and osteoarthritis outcome score; QoL, quality of life; PASS, patient‐acceptable symptomatic state; TAS, Tegner activity scale.

All of the six patients, who reported dissatisfaction after the surgery, were female. However, this was not statistically significant (*p* < 0.31).

### Comparison by age

When analysing the preoperative functional scores of our patients, there was no score that had any statistically significant correlation to the age at the time of surgery.

At 6 months follow‐up there were highly significant correlations to the age at the time of surgery in the **Kujala score** (*r* = −0.66, *p* = 0.02), the **KOOS ADL** (*r* = −0.57, *p* < 0.01), **KOOS QoL** (*r* = −0.39, *p* = 0.03), **KOOS pain** (*r* = −0.46, *p* = 0.01), **KOOS sport/recreation** (*r* = −0.53, *p* < 0.01) and **IKDC** (*r* = −0.52, *p* < 0.01) with negative correlations indicating significantly higher scores in younger patients. There were no significant differences in the **KOOS symptoms** (*r* = −0.30, *p* = 0.10) and **TAS** (*r* = −0.14, *p* = 0.50).

At 12 months follow‐up there were significant correlations in the **KOOS pain** (*r* = −0.50, *p* < 0.01), **KOOS ADL** (*r* = −0.52, *p* < 0.01), **KOOS sport/recreation** (*r* = −0.45, *p* = 0.02), **KOOS QoL** (*r* = −0.52, *p* < 0.01), the **IKDC** (*r* = −0.55, *p* < 0.01) and the **TAS** (*r* = −0.40, *p* = 0.04). There was no statistically significant correlation of the **Kujala score** (*r* = −0.54, *p* = 0.07) with age.

**Figure 2:**
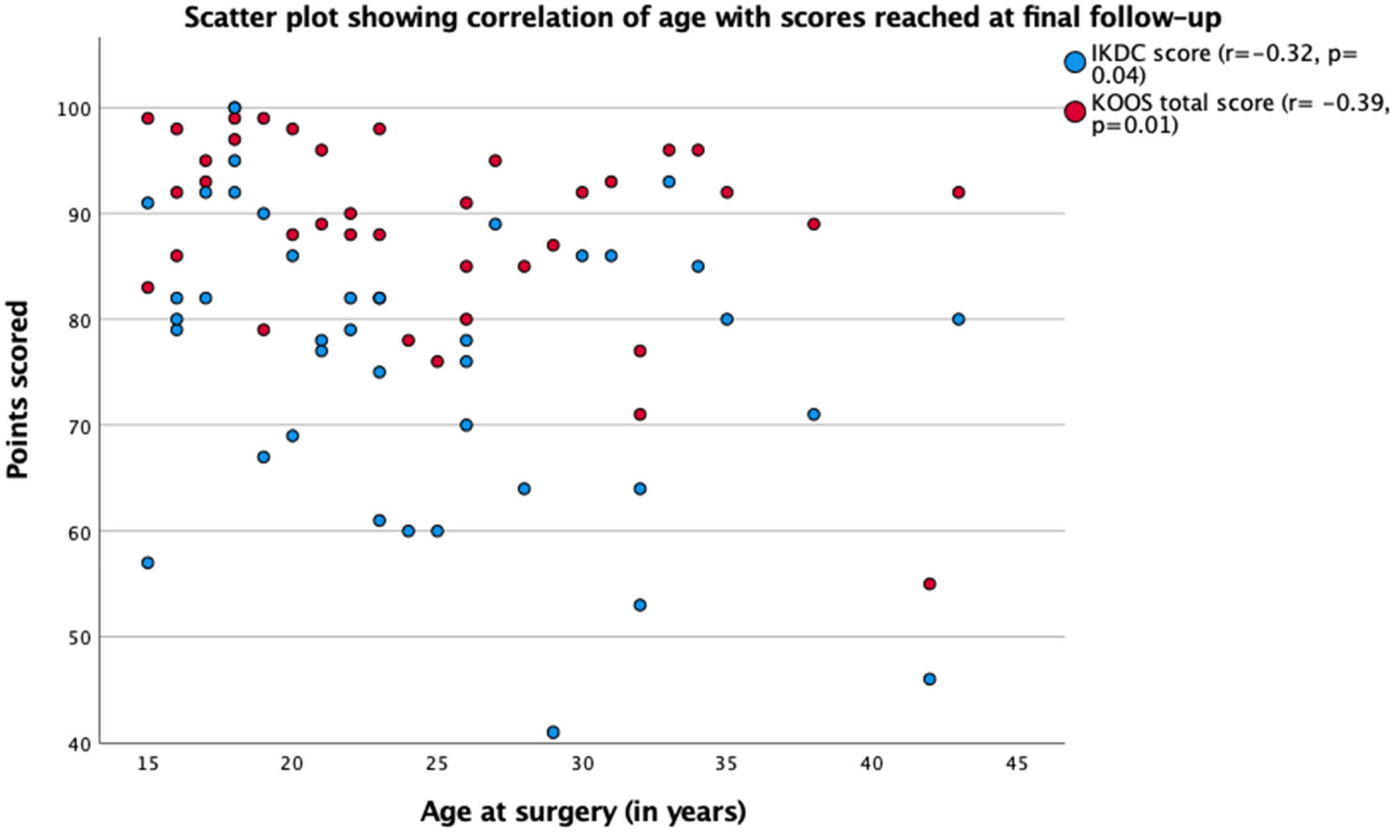
Scatter plot depicting the correlation of age with scores reached at final follow‐up exemplary for knee injury and osteoarthritis outcome score (KOOS) total score and International Knee Documentation Committee (IKDC) score at final follow‐up.

At final follow‐up, we found significant correlations of age with the **KOOS total** (*r* = −0.39, *p* = 0.01), **KOOS pain** (*r* = −0.36, *p* = 0.04), **KOOS ADL** (*r* = −0.33, *p* = 0.04), **KOOS sport/recreation** (*r* = −0.46, *p* ≤ 0.01), and **IKDC** (*r* = −0.32, *p* = 0.04) all show significant negative correlations, indicating worse outcomes with increasing age (Figure 2).

The **Kujala score** (*r* = −0.31, *p* = 0.06), **KOOS symptoms** (*r* = −0.27, *p* = 0.09), **KOOS QoL** (*r* = −0.19, *p* = 0.26), and the **TAS** (*r* = −0.17, *p* = 0.20) show weak to negligible significant correlations with age, indicating that higher age is associated with lower levels of activity and worse symptoms, however this is not statistically significant.

### Comparison by BMI

Preoperatively only the **KOOS sport/recreation** score showed a statistically significant correlation to BMI (*r* = −0.42, *p* < 0.01), indicating that patients with higher BMI had lower scores in the KOOS sport and recreation section.

At 6‐ and 12‐month follow‐up, there were also no statistically significant correlations between BMI and the functional outcome.

At final follow‐up there was also no statistically significant correlation between BMI and the **Kujala score** (*r* = 0.11, *p* = 0.53), **KOOS total score** (*r* = 0.07, *p* = 0.70), **KOOS pain** (*r* = 0.03, *p* = 0.89), **KOOS ADL** (*r* = 0.11, *p* = 0.53), and the **KOOS sport/recreation** (*r* = 0.11, *p* = 0.51), **KOOS QoL** (*r* = −0.02, *p* = 0.91), **KOOS symptoms** (*r* = −0.04, *p* = 0.82), **IKDC** (*r* = 0.05, *p* = 0.78) or **TAS** (*r* = 0.12, *p* = 0.38).

Also, when comparing the normal to the high BMI groups there were no significant differences in the functional outcome scores at final follow‐up. There was also no significant correlation to the percentage of patients reaching or exceeding PASS in these groups.

## DISCUSSION

The main finding of this study is, that age at surgery and not BMI or biological sex affects outcome after isolated MPFL reconstruction in patients with recurrent patellar dislocation in the absence bony risk factors.

Diagnostic and treatment algorithms for chronic patellar dislocations emphasise the importance of a thorough evaluation of patient‐related risk factors, such as bony incongruence, in devising an appropriate treatment plan [6, 18, 35]. Furthermore, it is well established that the presence of such deformities significantly impacts the choice of surgical intervention, postoperative management, and the anticipated outcomes [20]. By establishing this ‘clean’ study group, we effectively eliminated potential confounders for poorer outcomes and minimised selection bias.

Overall, our results demonstrate favourable clinical outcomes following isolated MPFL reconstruction, with a significant proportion of patients exceeding the PASS threshold by the final follow‐up. The outcomes in our study were comparable to those reported in a systematic review, which documented a mean Kujala score of 85.8, closely aligning with the 87.0 observed in our cohort. Similarly, the Tegner score in the review averaged 5.7, compared to 5.1 in our study [21]. In our study group, four patients experienced a redislocation, resulting in a redislocation rate of 6.5%. This rate falls within the range of recurrent dislocation reported in the existing literature [11, 21]. Runer et al. also reported similar rate of patients to exceed the PASS in the Kujala score of 68.8%, when an autologous hamstring graft was used for MPFL reconstruction [18]. In the present cohort, 96.7% of the patients were treated with an autologous hamstring graft.

Our study primarily aimed to investigate the influence of patient‐specific factors—namely age, sex, and BMI—on outcomes following MPFL reconstruction. We observed a significant negative correlation between increasing age and functional outcomes, as reflected in the scoring systems. Although not statistically significant in some categories (Kujala score, KOOS symptoms, KOOS QoL and TAS) at final follow‐up, this negative trend was consistently observed across all scoring systems. As seen in Figure 2, some younger patients also presented with lower IKDC scores at final follow‐up, suggesting that age alone is not the sole determinant of outcome variability following MPFL reconstruction surgery. However, higher age still has a statistically significant correlation with IKDC levels.

Our findings align with those of another study group, who also identified older age as a negative predictor of functional outcomes after MPFL reconstruction [10].

Several factors may explain why age was a relevant determinant of functional outcomes in our study cohort. Patient‐reported outcomes are influenced by various dimensions of patient satisfaction, including emotional well‐being, the ability to engage in desired sports and recreational activities, pain tolerance in daily life, and the fear of redislocation. Given the multidimensional nature of patient satisfaction, we opted to use a range of scores to assess functional outcomes following MPFL reconstruction comprehensively. The KOOS and IKDC scores were employed as general knee assessment tools, while the Kujala and BANFF scores, which are specifically designed for anterior knee pain and chronic patellofemoral instability, provided more targeted insights into this condition [4, 5, 30]. They have been translated into German, are validated, and are widely used for research purposes [17, 31].

Considering this, a longer duration between the initial dislocation and surgery—leading to an older age at the time of surgery—might indicate that patients were unable to perform at their optimal level for an extended period. Chronic misalignments or compensatory postures developed during this time could result in poorer biomechanical adaptations, ultimately hindering the return to the desired activity levels and quality of life. However, in this study, we assessed age at the time of surgery rather than at the time of the initial dislocation. Additionally, osteoarthritis and early cartilage damage, which are common in patients with recurrent patellar dislocations or those engaged in high levels of sports activity from a young age, could also contribute to poorer outcomes associated with older age at surgery, however, the presence of high‐grade chondral injuries and osteoarthritis served as an exclusion criterion in our study. On a pathophysiological level, the regenerative capacity of tissues diminishes significantly with advancing age, further explaining the observed impact of age on functional outcomes [9].

These results warrant further discussion into what the ideal time for surgery after patellar dislocation is. Multiple studies have suggested that very young age at first patellar dislocation is a predictor for chronic instability [7, 13]. This is attested partially to the fact that high‐grade bony risk factors might present themselves early on, and early surgery is well regarded in these cases [12, 33]. On the other hand, an immature skeleton might hinder or even prevent surgeons from performing necessary bony alignment surgery such as trochlear plasty surgery, rendering isolated soft tissue fixation rather than reconstruction unhelpful [8]. To date there is no literature that would warrant general early MPFL reconstruction or surgical treatment of all first‐time patellar dislocations. However, recent reviews showed that MPFL reconstruction has superior clinical outcomes compared to conservative treatment [12, 27]. One aspect, which could also explain some of the low outlier scores in younger patients, is the relevance of psychological factors in recovery and perioperative treatment in patients with chronic patellofemoral instability. This factor has found increasing attention in recent years [19].

Overall, we observe that the oldest patients tend to have the lowest outcomes across nearly all measured scores. Therefore, it is important to emphasise that surgeons should exercise extra caution and may even consider avoiding surgery in these older patients. A clear cut‐off point for patients age cannot be defined with our study, but further research should be conducted to evaluate possible guidelines.

As mentioned before, sex specific differences have found increasing attention in recent years. While anatomical differences have been well evaluated in the knee, the influence of sex for the outcome after isolated MPFL reconstruction is controversially discussed [10, 16, 21, 28].

The results of our study indicate that biological sex does not correlate with functional outcomes following isolated MPFL reconstruction. This finding aligns with the results reported by several other authors [10, 13]. In contrast, Parikh et al., in their retrospective study, reported a higher complication rate, including increased recurrence of instability and impaired range of motion. However, their study did not differentiate patients with high‐grade bony risk factors, which may have influenced their findings [16]. Of the four patients in our study who experienced a redislocation, three were female. Furthermore, all six patients who reported dissatisfaction with the surgical outcomes were also female. However, the small sample size limits generalisation and differences were not statistically significant. Nevertheless, it is important to consider these health‐related quality of life aspects and their differences between men and women [1]. Some aspects of this are already addresses in several PROMs, including those used in this study, like the BANFF or KOOS score.

This study has several limitations. As mentioned above, the sample size was too small to establish a statistically significant correlation between dissatisfaction and biological sex. Some of the statistically significant correlations only found weak correlation. Additionally, the retrospective design has inherent limitations, particularly in assessing the time from the first dislocation or subluxation to surgery, as patients' responses were often vague and unreliable and the cause for re‐dislocation could not be determined accurately. Patients were not assessed in person, meaning functional outcomes were measured solely through patient‐reported scores.

## CONCLUSION

Patient‐reported outcomes after isolated MPFL reconstruction in patellofemoral instability are influenced by patients' age at the time of surgery. Abnormal BMI and the biological sex seem to have no statistically significant impact on the outcome. Female patients seem to be dissatisfied with surgical results more frequently than men. Surgeons must be highly vigilant and identify high‐risk patients even before surgery in order to time necessary MPFL reconstruction appropriately.

## AUTHOR CONTRIBUTIONS

**Olivia Bohe**: Collection of data; statistical analysis of data; writing of manuscript. **Antonia Schneider**: Collection of data; data handling; statistical analysis of data; correction of manuscript. **Armin Runer**: Correcting of the manuscript; supervision. **Sebastian Siebenlist**: Conception of study; supervision; correction of manuscript. **Andrea Achtnich**: Conception of study; supervision of data collection and interpretation of data; writing of manuscript. All authors read and approved the submitted version of the manuscript.

## CONFLICT OF INTEREST STATEMENT

The authors declare no conflicts of interest.

## ETHICS STATEMENT

Approval by the ethics committee of the Technical University Munich was obtained (Study No 2022‐193‐S‐NP). The study complied with the Declaration of Helsinki and its respective amendments. Informed consent was obtained from all patients prior to participation in this study.

## Data Availability

The datasets used and/or analysed during the current study are available from the corresponding author on reasonable request.

## References

[1] Balcarek P , Milinkovic DD , Zimmerer A , Zimmermann F . Mental and physical health‐related quality of life in patients with recurrent patellar dislocations: a generic and disease‐specific quality of life questionnaire assessment. J Exp Orthop. 2022;9:60. 35764849 10.1186/s40634-022-00499-3 PMC9240127

[2] Balcarek P , Oberthür S , Hopfensitz S , Frosch S , Walde TA , Wachowski MM , et al. Which patellae are likely to redislocate? Knee Surg Sports Traumatol Arthrosc. 2014;22:2308–2314. 24005331 10.1007/s00167-013-2650-5

[3] Conley S , Rosenberg A , Crowninshield R . The female knee: anatomic variations. J Am Acad Orthop Surg. 2007;15(Suppl 1):S31–S36. 17766787 10.5435/00124635-200700001-00009

[4] Crossley KM , Bennell KL , Cowan SM , Green S . Analysis of outcome measures for persons with patellofemoral pain: which are reliable and valid? Arch Phys Med Rehabil. 2004;85:815–822. 15129407 10.1016/s0003-9993(03)00613-0

[5] Dammerer D , Liebensteiner MC , Kujala UM , Emmanuel K , Kopf S , Dirisamer F , et al. Validation of the German version of the Kujala score in patients with patellofemoral instability: a prospective multi‐centre study. Arch Orthop Trauma Surg. 2018;138:527–535. 29372384 10.1007/s00402-018-2881-5 PMC5854722

[6] Erickson BJ , Nguyen J , Gasik K , Gruber S , Brady J , Shubin Stein BE . Isolated medial patellofemoral ligament reconstruction for patellar instability regardless of tibial tubercle‐trochlear groove distance and patellar height: outcomes at 1 and 2 years. Am J Sports Med. 2019;47:1331–1337. 30986090 10.1177/0363546519835800

[7] Fithian DC , Paxton EW , Stone ML , Silva P , Davis DK , Elias DA , et al. Epidemiology and natural history of acute patellar dislocation. Am J Sports Med. 2004;32:1114–1121. 15262631 10.1177/0363546503260788

[8] Gao B , Dwivedi S , Fabricant PD , Cruz Jr. AI Patterns in outcomes reporting of operatively managed pediatric patellofemoral instability: a systematic review and meta‐analysis. Am J Sports Med. 2019;47:1516–1524. 29630397 10.1177/0363546518765152

[9] Hamilton DF , McLeish JA , Gaston P , Simpson AHRW . Muscle ‘regenerative potential’ determinesphysical recovery following total knee replacement. Bone Jt Res. 2013;2:70–78. 10.1302/2046-3758.24.2000151 PMC3638306 23673375

[10] Hiemstra LA , Kerslake S . Age at time of surgery but not sex is related to outcomes after medial patellofemoral ligament reconstruction. Am J Sports Med. 2019;47:1638–1644. 31063706 10.1177/0363546519841371

[11] Jackson GR , Tuthill T , Gopinatth V , Mameri ES , Jawanda H , Sugrañes J , et al. Complication rates after medial patellofemoral ligament reconstruction range from 0% to 32% with 0% to 11% recurrent instability: a systematic review. Arthrosc J Arthrosc Rel Surg. 2023;39:1345–1356. 10.1016/j.arthro.2023.01.098 36764559

[12] Le N , Blackman B , Zakharia A , Cohen D , de Sa D . MPFL repair after acute first‐time patellar dislocation results in lower redislocation rates and less knee pain compared to rehabilitation: a systematic review and meta‐analysis. Knee Surg Sports Traumatol Arthrosc. 2023;31:2772–2783. 36372845 10.1007/s00167-022-07222-w

[13] Lewallen L , McIntosh A , Dahm D . First‐time patellofemoral dislocation: risk factors for recurrent instability. J Knee Surg. 2015;28:303–310. 25633361 10.1055/s-0034-1398373

[14] Merchant AC , Arendt EA , Dye SF , Fredericson M , Grelsamer RP , Leadbetter WB , et al. The female knee: anatomic variations and the female‐specific total knee design. Clin Orthop Rel Res. 2008;466:3059–3065. 10.1007/s11999-008-0536-5 PMC2592531 18820981

[15] Mitchell J , Magnussen RA , Collins CL , Currie DW , Best TM , Comstock RD , et al. Epidemiology of patellofemoral instability injuries among high school athletes in the United States. Am J Sports Med. 2015;43:1676–1682. 25899431 10.1177/0363546515577786

[16] Parikh SN , Nathan ST , Wall EJ , Eismann EA . Complications of medial patellofemoral ligament reconstruction in young patients. Am J Sports Med. 2013;41:1030–1038. 23539043 10.1177/0363546513482085

[17] Roos EM , Roos HP , Lohmander LS , Ekdahl C , Beynnon BD . Knee injury and osteoarthritis outcome score (KOOS)—development of a self‐administered outcome measure. J Orthop Sports Phys Ther. 1998;28:88–96. 9699158 10.2519/jospt.1998.28.2.88

[18] Runer A , Klotz S , Schneider F , Egelseer T , Csapo R , Hoser C , et al. Medial patellofemoral ligament reconstruction using pedicled quadriceps tendon autograft yields similar clinical and patient‐reported outcomes but less donor‐site morbidity compared with gracilis tendon autograft. Arthrosc J Arthrosc Rel Surg. 2024;40:438–445. 10.1016/j.arthro.2023.07.006 37479150

[19] Ryan PC , Ching IC , Ierulli VK , Pickett K , Mulcahey MK . Fear of reinjury, psychological factors, and sport played have negative impact on return to sport following medial patellofemoral ligament reconstruction for patellar instability. Arthroscopy 2025;41(5):1605–1617. 38849062 10.1016/j.arthro.2024.05.022

[20] Sappey‐Marinier E , Sonnery‐Cottet B , O'Loughlin P , Ouanezar H , Reina Fernandes L , Kouevidjin B , et al. Clinical outcomes and predictive factors for failure with isolated MPFL reconstruction for recurrent patellar instability: a series of 211 reconstructions with a minimum follow‐up of 3 years. Am J Sports Med. 2019;47:1323–1330. 31042437 10.1177/0363546519838405

[21] Schneider DK , Grawe B , Magnussen RA , Ceasar A , Parikh SN , Wall EJ , et al. Outcomes after isolated medial patellofemoral ligament reconstruction for the treatment of recurrent lateral patellar dislocations: a systematic review and meta‐analysis. Am J Sports Med. 2016;44:2993–3005. 26872895 10.1177/0363546515624673 PMC5502077

[22] Schober P , Boer C , Schwarte LA . Correlation coefficients: appropriate use and interpretation. Anesth Analg. 2018;126:1763–1768. 29481436 10.1213/ANE.0000000000002864

[23] Senavongse W , Amis AA . The effects of articular, retinacular, or muscular deficiencies on patellofemoral joint stability: a biomechanical study in vitro. J Bone Jt Surg Br. 2005;87:577–582. 10.1302/0301-620X.87B4.14768 15795215

[24] Sharma N , Al‐Mouazzen L , Kuiper JH , Gallacher P , Barnett A . Functional outcomes after medial patellofemoral ligament reconstruction show inverted J‐shaped relation with body mass index. Knee Surg Sports Traumatol Arthrosc. 2023;31:3381–3389. 37036473 10.1007/s00167-023-07391-2

[25] Sherman SL , Rund JM , Welsh JW , Ray T , Worley JR , Oladeji LO , et al. Medial patellofemoral ligament reconstruction in obese patients results in low complication rates and improved subjective outcomes. Arthrosc Sports Med Rehabil. 2023;5:e257–e262. 36866317 10.1016/j.asmr.2022.11.023 PMC9971998

[26] Stupay KL , Swart E , Shubin Stein BE . Widespread implementation of medial patellofemoral ligament reconstruction for recurrent patellar instability maintains functional outcomes at midterm to long‐term follow‐up while decreasing complication rates: a systematic review. Arthrosc J Arthrosc Rel Surg. 2015;31:1372–1380. 10.1016/j.arthro.2014.12.029 25703288

[27] Tian G , Yang G , Zuo L , Li F , Wang F . Conservative versus repair of medial patellofemoral ligament for the treatment of patients with acute primary patellar dislocations: a systematic review and meta‐analysis. J Orthop Surg. 2020;28:2309499020932375. 10.1177/2309499020932375 32552381

[28] van Sambeeck JDP , Verdonschot N , Van Kampen A , van de Groes SAW . Age at surgery is correlated with pain scores following trochlear osteotomy in lateral patellar instability: a cross‐sectional study of 113 cases. J Orthop Surg. 2021;16:337. 10.1186/s13018-021-02485-4 PMC8146238 34034788

[29] Walsh JM , Huddleston HP , Alzein MM , Wong SE , Forsythe B , Verma NN , et al. The minimal clinically important difference, substantial clinical benefit, and patient‐acceptable symptomatic state after medial patellofemoral ligament reconstruction. Arthrosc Sports Med Rehabil. 2022;4:e661–e678. 35494257 10.1016/j.asmr.2021.12.009 PMC9042905

[30] Watson CJ , Propps M , Ratner J , Zeigler DL , Horton P , Smith SS . Reliability and responsiveness of the lower extremity functional scale and the anterior knee pain scale in patients with anterior knee pain. J Orthop Sports Phys Ther. 2005;35:136–146. 15839307 10.2519/jospt.2005.35.3.136

[31] Wright RW . Knee injury outcomes measures. J Am Acad Orthop Surg. 2009;17:31–39. 19136425 10.5435/00124635-200901000-00005

[32] Zhan H , Liu J , Sheng X , Yi Z , Feng Z , Wang Q , et al. Comparison of clinical and radiological outcomes with body mass index after medial patellofemoral ligament reconstruction. Orthop J Sports Med. 2024;12:23259671241270358. 39473767 10.1177/23259671241270358 PMC11520002

[33] Zhang GY , Ding HY , Li EM , Zheng L , Bai ZW , Shi H , et al. Incidence of second‐time lateral patellar dislocation is associated with anatomic factors, age and injury patterns of medial patellofemoral ligament in first‐time lateral patellar dislocation: a prospective magnetic resonance imaging study with 5‐year follow‐up. Knee Surg Sports Traumatol Arthrosc ESSKA. 2019;27:197–205. 10.1007/s00167-018-5062-8 30008056

[34] Zheng ET , Kocher MS , Wilson BR , Hussain ZB , Nunally KD , Yen YM , et al. Descriptive epidemiology of a surgical patellofemoral instability population of 492 patients. Orthop J Sports Med. 2022;10:23259671221108174. 35859643 10.1177/23259671221108174 PMC9289910

[35] Zimmermann F , Milinkovic DD , Zimmerer A , Balcarek P . When should bony correction be considered in addition to medial patellofemoral ligament reconstruction? results of a clinically derived 2‐group classification of lateral patellar instability based on 122 patients at 2‐ to 5‐year follow‐up. Orthop J Sports Med. 2023;11:23259671221147572. 36743734 10.1177/23259671221147572 PMC9893382

